# Vestibulo-ocular monitoring as a predictor of outcome after severe traumatic brain injury

**DOI:** 10.1186/cc8187

**Published:** 2009-11-30

**Authors:** Hans-Georg Schlosser, Jan-Nikolaus Lindemann, Peter Vajkoczy, Andrew H Clarke

**Affiliations:** 1 Department of Neurosurgery, Universitätsmedizin Berlin, Charité - Campus Virchow Klinikum, Augustenburger Platz 1, Berlin 13353, Germany; 2 Institute of Physiology, Universitätsmedizin Berlin, Charité - Campus Benjamin Franklin, Arnimallee 22, Berlin 14195, Germany; 3 ENT - Vestibular Research Laboratory, Universitätsmedizin Berlin, Charité - Campus Benjamin Franklin, Hindenburgdamm 30, Berlin 12200, Germany

## Abstract

**Introduction:**

Based on the knowledge that traumatic brainstem damage often leads to alteration in brainstem functions, including the vestibulo-ocular reflex, the present study is designed to determine whether prediction of outcome in the early phase after severe traumatic brain injury is possible by means of vestibulo-ocular monitoring.

**Methods:**

Vestibulo-ocular monitoring is based on video-oculographic recording of eye movements during galvanic labyrinth polarization. The integrity of vestibulo-ocular reflex is determined from the eye movement response during vestibular galvanic labyrinth polarization stimulation. Vestibulo-ocular monitoring is performed within three days after traumatic brain injury and the oculomotor response compared to outcome after six months (Glasgow Outcome Score).

**Results:**

Twenty-seven patients underwent vestibulo-ocular monitoring within three days after severe traumatic brain injury. One patient was excluded from the study. In 16 patients oculomotor response was induced, in the remaining 11 patients no oculomotor response was observed. The patients' outcome was classified as Glasgow Outcome Score 1-2 or as Glasgow Outcome Score 3 to 5. Statistical testing supported the hypothesis that those patients with oculomotor response tended to recover (exact two-sided Fisher-Test (*P *< 10-3)).

**Conclusions:**

The results indicate that vestibulo-ocular monitoring with galvanic labyrinth polarization performed during the first days after traumatic brain injury helps to predict favourable or unfavourable outcome. As an indicator of brainstem function, vestibulo-ocular monitoring provides a useful, complementary approach to the identification of brainstem lesions by imaging techniques.

## Introduction

Severe traumatic brain injury (sTBI) is the most prevalent cause of mortality and severe morbidity in young adults in industrialized countries, for example, in Germany 30,000 people suffer from severe brain trauma each year. A total of 10,000 result in death, and a further 4,500 have a severe disabled outcome and require permanent care (Federal Statistic Office). At present, assessment of outcome in the acute phase of sTBI is difficult and the contributing elements are under discussion.

One promising approach to improving this situation has been the examination of the brainstem using imaging techniques. This has permitted classification of the extent of brainstem lesions in sTBI and association of different categories with outcome [1-3].

In the present study we have introduced vestibulo-oculomotor monitoring (VOM) as a means of testing brainstem function. This is intended to complement the findings of brainstem imaging and thus should improve the prognostication of long-term outcome.

In principle, the proposed vestibulo-ocular monitoring technique is based on video-oculographic (VOG) recording of eye movements during galvanic labyrinth polarization (GaLa) stimulation of both labyrinths. The eye movement response is elicited via the vestibulo-ocular reflex arc (VOR), that is, via the afferents from the peripheral neurons to the vestibular nuclei and subsequently to the oculomotor neurons.

VOM as a means of examining comatose patients was introduced [4] and equipment suitable for use with patients in the neuro intensive care unit (nicu) suffering from sTBI was developed.

The aim of the present study was to perform VOM in the intensive care unit during the acute phase of sTBI. Oculomotor response (OMR), as elicited by GaLa, was recorded and analysed and the extent to which these correlated with the six-month outcome (GOS) was determined. The feasibility of performing such measurements in the intensive care unit is discussed and the question as to whether the technique is useful for the prognosis of patient outcome is examined.

## Materials and methods

Patients admitted to the Charité University Hospital neurocritical care unit (nicu) for therapy of severe traumatic brain injury (sTBI) were included in the study. The cause of sTBI accumulates as falls (n = 6), bicycle accidents (n = 5), pedestrian accidents (n = 4), car accidents (n = 4) and motor-cycle accidents (n = 2). Further clinical data is presented in Table 1. The following criteria were applied for their selection. Patients were required to initially score less than nine points on the initial Glasgow Coma Score (GCS), and had to be intubated and ventilated. It was further required that the computer tomography (CT) scan performed on admission had to show signs of traumatic brain injury. The patients did not suffer from isolated brain injuries alone. For this reason The Acute Physiology And Chronic Health Evaluation II Score (APACHE II) and Simplified Acute Physiology Score II (SAPS II) Score were taken to assess the extent of the trauma to the whole body. The patients' records of medication and intracranial pressure (icp) during their stay and during the VOM procedure were stored in the electronic patient documentation system of the intensive care unit.

**Table 1:** Clinical Data

Group	Number of Patients	Neurological Status 6 months	Mean Age	APACHE II (mean)	Emergency surgery before VOM
1	13	GOS < 3	45,4	17,9	7

2	13	GOS > 2	38,3	22,1	6

Patients were classified into two groups according to the Glasgow Outcome Scale (GOS) at six months post-trauma. In *emergency surgery *before performing Vestibulo-ocular monitoring (VOM) evacuation of epidural-, subdural haematoma or contusion was integrated. The implantation of intracranial pressure (icp) measurement or ventricular drainage is not included in *emergency surgery*.

GOS = Glasgow Outcome Scale; icp = intracranial pressure; VOM = vestibulo-ocular monitoring

**Figure 1:**
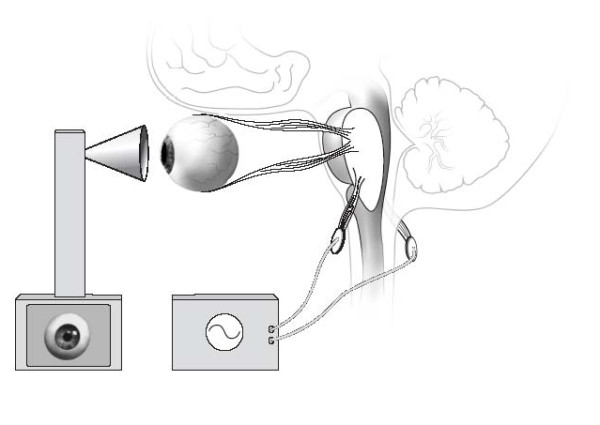
Vestibulo-ocular monitoring consisting of galvanic labyrinth polarization and video-oculography. The two components of vestibulo-ocular monitoring are depicted: Galvanic labyrinth polarization as a vestibular stimulus to the vestibular nerve; video-oculography recording of eye movement in response to the Galvanic labyrinth polarization stimulus.

**Figure 2:**
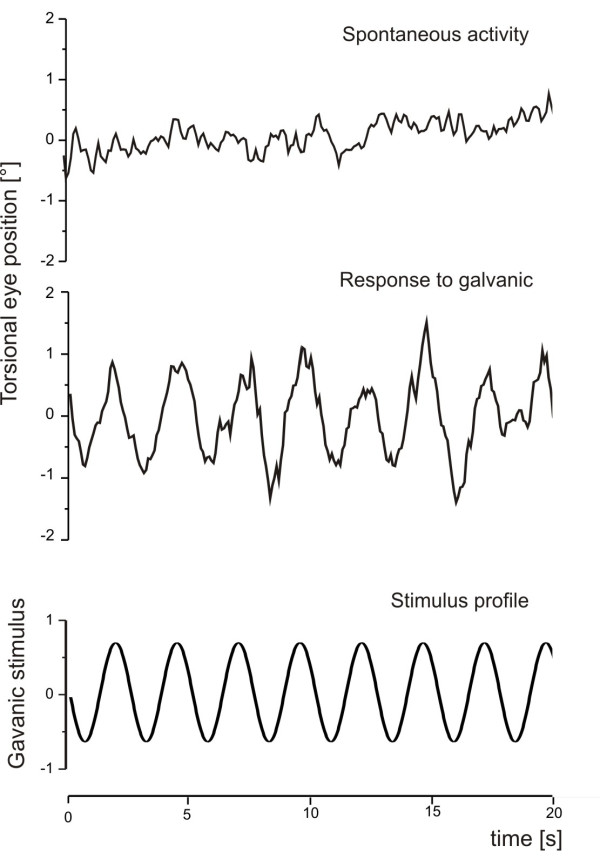
Oculomotor response in a healthy volunteer. Original recordings of oculomotor response in a healthy volunteer depicting spontaneous oculomotor response and an oculomotor response induced by Galvanic labyrinth polarization.

Vestibulo-ocular monitoring (VOM) was performed within the first three days after trauma. At this stage all patients were still intubated and ventilated. Thus, GaLa was applied to elicit a vestibulo-ocular response; eye movements were recorded throughout by videooculography (Figures 1 and 2). Each examination consisted of recording one minute of spontaneous eye movement (no stimulation) and a second one-minute period with GaLa stimulation.

The GaLa was applied via circular silverchloride adhesive surface electrodes of 50 mm diameter, attached to the right and left mastoids. A further electrode was attached interscapular. Stimulation was applied respectively between the right and the left electrode pairs each pair consisting of an electrode over the mastoid and interscapular. Accordingly, independent stimulation of the right or the left labyrinths was possible.

The term galvanic labyrinth polarization (GaLa) was originally introduced after the discovery of *animal electricity *by Galvani [5] and is still employed, despite the fact that the galvanic stimulation acts on the postsynaptic membrane in the vestibular nerve rather than the receptors in the labyrinths as first supposed [6-8]. Thus the afferents from all three semicircular canals and from the two otolith organs are stimulated. Galvanic stimulation thus facilitates systematic examination of the vestibular response by employing sinusoidal modulated current of defined amplitude and frequency [9-11]. A custom-manufactured galvanic stimulator (Neurotronix, Berlin, Germany) generated the required current output. This was set so that the currents to the right and left of each labyrinths were 180 degrees out of phase. Thus, one labyrinth was polarized maximally, while the other was in the opposite direction at a minimum. A sinusoidal waveform of 0.41 Hz and a current level of 8 mA were employed. This stimulus level was chosen on the basis of control experiments which demonstrated that this level elicited a response in all tested volunteer subjects [12]. In addition, a previous study [4] has shown that GaLa stimulus frequencies in the range 0.35 to 2.0 Hz induce OMR [4,12]. The frequency employed here was selected in this range and with a value that excluded any harmonic effects from possible interference signals in the nicu. The stimulus profile was recorded together with the OMR.

**Figure 3:**
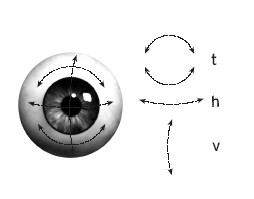
Components of the oculomotor response. The three components of the oculomotor response are depicted: t = torsional movement, h = horizontal movement, v = vertical movement.

Eye movements were recorded by videooculography (VOG). This consisted of a head-fixed camera system mounted in a modified set of goggles. The right eye was recorded at a frame rate of 50 Hz by an infrared eye tracker (Chronos Vision, Berlin, Germany). The resulting digital image sequences were recorded on hard disk and analyzed off-line. Three-dimensional eye position (torsional, vertical, horizontal, see Figure 3) was computed for each frame using the IRIS software package (Chronos Vision). The eye movement data provided the basis for determining the extent of a stimulus-dependent response. The Origin™V8.0 software package (OriginLab Corporation, Northampton, USA) was employed to perform frequency analysis (FFT) on the resultant torsional, horizontal and vertical components of the eye movement records. Essentially, the spectra of the eye movement components during spontaneous activity and during GaLa were compared. When the OMR power spectrum showed a clear peak at the frequency of the stimulus, the patient was classified as a responder.

Each patient's outcome was evaluated using a structured interview according to the GOS. To this end the patient or the caretaker was questioned in the outpatient clinic or per telephone interview six months after the trauma.

The statistical analysis for the exact two-sided Fisher test for cross-tables (SPSS version 12.0.1 g SPSS Inc., Chicago, USA) was employed to test for any correlation between OMR and GOS.

The study was approved by the Ethics Committee of the Charité Medical School and performed in accordance with National Institute of Health guidelines. Informed consent was given by the patient's legal guardian.

## Results

VOM was performed within the first three days after sTBI in 27 patients (22 males - 81.5%, and 5 females - 18.5%). All patients had an initial GCS lower than nine at the place of accident, and had been intubated and ventilated by the emergency physician. Mean age was 44.6 years. All patients were treated according to standard guidelines. All patients showed a structural lesion in CT scans due to the trauma (see Table 2). Cases with ocular or orbital lesions, which could influence the OMR, were excluded from the study. All patients were intubated and ventilated when VOM was applied. In 26 of the 27 patients included in the study, a follow-up examination was performed six months after trauma.

**Table 2:** Computer tomography findings, medication, oculomotor response

Outcome	OM	CCT	Sedative Medication
GOS<3	induced (n = 2)	Contusion, Subdural haematoma, Traumatic subarachnoid haemorrhage, skull fracture	Fentanyl, remifentanyl, midazolam, propofol, thiopental
	
	Not induced (n = 11)	Contusion, Subdural haematoma, Epidural haematoma, Traumatic subarachnoid haemorrhage, skull fracture, skull base fracture, Spine fracture	Fentanyl, remifentanyl, midazolam, propofol, esketamin, thiopental

GOS>2	induced (n = 13)	Contusion, Subdural haematoma, Epidural haematoma, Traumatic subarachnoid haemorrhage, skull fracture, skull base fracture, spine fracture	Fentanyl, remifentanyl, midazolam, propofol, ketamin, esketamin, thiopental, clonidin, methohexital
	
	Not induced (n = 0)		

According to outcome and OMR, the structural lesions in CCT and the sedative medication are listed.

CCT = computer tomography; OMR = oculomotor response

The APACHE II and SAPS were employed to evaluate physiological status. The mean APACHE II score was 20.9 and mean SAPS was 45.3. Those patients classified as GOS <3 had a mean APACHE II score of 17.9 and a mean age of 45.4; the mean APACHE II score for those classified as GOS >2 was 22.1, and their mean age was 38.3.

Of the 27 patients included, one died of multi-organ failure in the acute phase as a result of his concomitant injuries. This patient was excluded from further evaluation.

VOM was performed in 23 patients (85.2%) during administration of sedative therapy. Combined therapy of fentanyl, remifentanyl, midazolam, propofol, ketamin, esketamin, thiopental, clonidin or methohexital was used according to clinical needs. Details on sedative therapy and OMR are given in Table 2. In one patient, who had received rocuroniumbromid for muscle relaxation, no OMR was observed initially. However, after medication had been discontinued, a GaLa induced OMR could be recorded.

In a further 10 patients with whom VOM was repeated as a control, consistent results were obtained.

**Figure 4:**
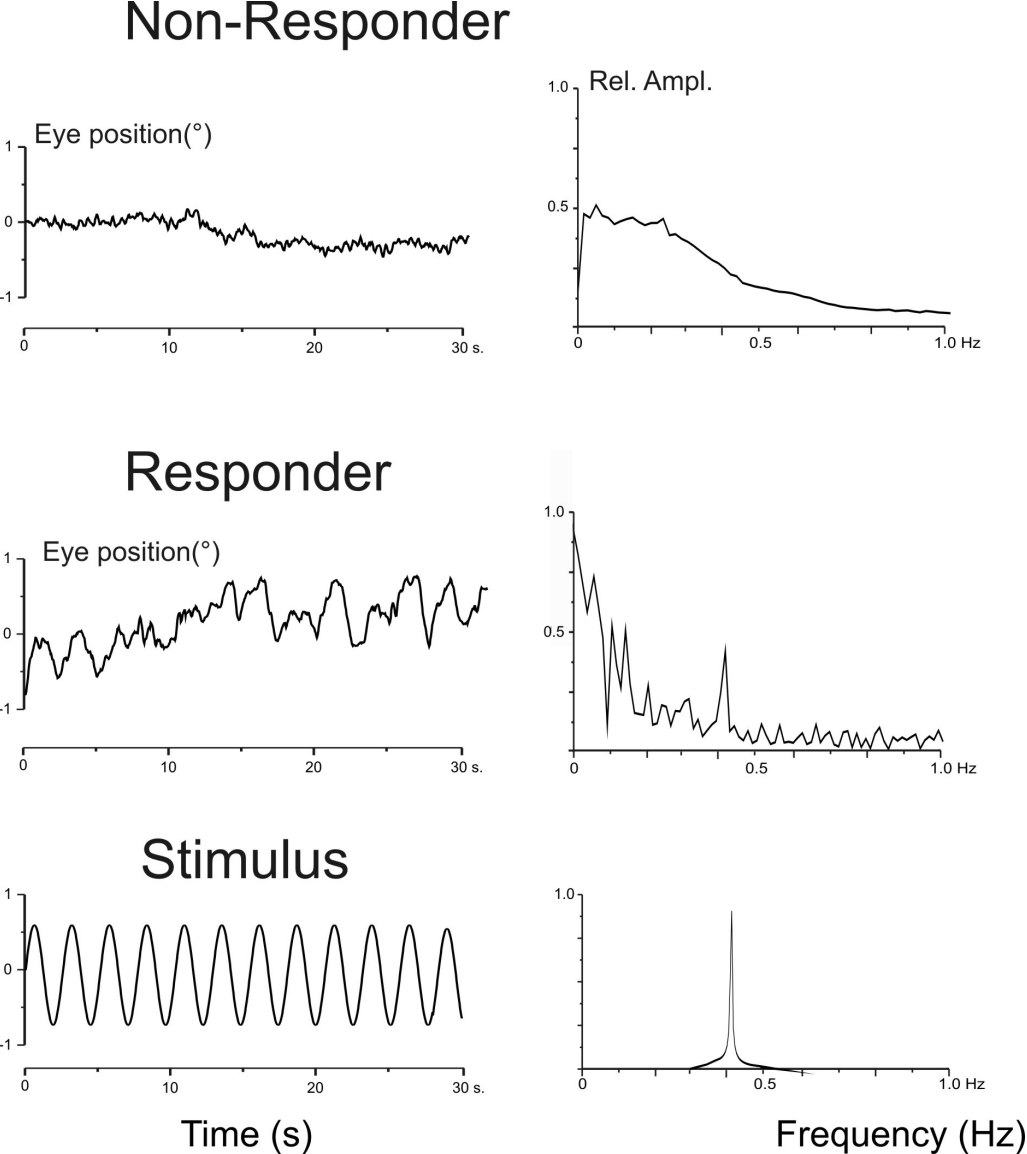
Oculomotor response and spectral analysis. Left: Original recordings of oculomotor response in two patients together with the sine wave stimulus (first column). Right: Corresponding frequency spectra of the oculomotor response and stimulus. The first patient failed to show an oculomotor response, that is, no response during Galvanic labyrinth polarization stimulation. The Glasgow Outcome Score after six months was 1. The second patient showed an oculomotor response to Galvanic labyrinth polarization stimulation: The frequency spectrum reflects the oculomotor response component the stimulus frequecy (0.41 Hz) in synchrony with the Galvanic labyrinth polarization stimulus. This patient survived with an Glasgow Outcome Score of 4.

From the total of 26 patients, GaLa induced eye movement responses were recorded in 15 (57.7%) (see Table 3 (GOS)). In the remaining 11 cases (42.3%) no OMR could be induced (Figures 4 and 2). In this latter group, all patients had an unfavourable outcome (GOS <3). Ten of these patients (90.9%) died. All died within 15 days after trauma (mean 7.0 days), six patients were operated on to reduce intracranial pressure for subdural hematoma, epidural hematoma or contusion additional to intraventricular drainage.

**Table 3:** Relationship between oculomotor response and Glasgow Outcome Score

OM			
**GOS**	**induced**	**not induced**	**Sum**

1	**0**	**10**	10

2	**2**	**1**	3

3	**5**	**0**	5

4	**6**	**0**	6

5	**2**	**0**	2

Sum	15	11	26

OMR = Oculomotor response

GOS = Glasgow Outcome Score

From the first group showing eye movement responses (n = 15) 13 (86.7%) patients had an outcome of GOS >/= 3. An unfavourable outcome was determined in the other two patients. Thus, a prognosis with a GOS of less than three, but better than two (see Table 4 - cross-tabulation), is possible on the basis of the presence/absence of induced OMR. This was tested with the exact two-sided Fisher-Test (*P *< 0.001).

**Table 4:** Cross-Tabulation of oculomotor response and outcome

	GOS		
	
	<3	>2	Sum
No OM induced	**11**	**0**	11

OM induced	**2**	**13**	15

Sum	13	13	26

The outcome after distinguishing the two patient groups of Glasgow Outcome Score (GOS) 1-2 and GOS 3-5. These two groups are confronted with the results of Vestibulo-ocular monitoring (VOM) as OMR.

OMR = Oculomotor response

GOS = Glasgow Outcome Score

VOM = Vestibulo-ocular monitoring

As a basic clinical parameter of outcome, pupillary diameter was estimated in the patients at the same point in time as VOM was performed. In three patients the pupil was dilated (11.5%), in the other 23 the pupil size was myotic or normal (88.5%). All three patients with dilated pupils had unfavourable outcomes. Due to intubation and ventilation combined with analgentic and sedative medication during the acute phase after sTBI in all patients, no further detailed clinical evaluation was possible.

## Discussion

This is the first report of the use of VOM as an indicator of outcome prognosis in sTBI. For the two groups GOS 3 to 5 or GOS 1 to 2, prediction of the individual patients outcome - based on the GaLa-OMR criterion (that is, OMR induced or no OMR induced) - was possible (*P *< 0.001). This finding appears to be independent of administration of sedative therapy (see Table 2, demonstrating that sedative therapy was applied to all patients, regardless of whether they showed an OMR). In contrast, muscle relaxants can mask the effect of VOM. Apparently the extraocular muscles become paretic when muscle relaxants are administered, as was found in one patient. VOM in its components GaLa and VOG is a commercially available technology, which could be employed in any clinic. In further steps the use of VOM as a compact bedside test with ad hoc results is desirable. Here the automated analysis of OMR and simplification of hardware could lead to a new point-of-care technology.

In comparison to GaLa induced OMR, pupillary dilation is often employed as a predictor of an unfavourable outcome. However this parameter is associated with a very low sensitivity (*P *= 0.2). In the present study all patients with a dilated pupil also failed to show an OMR. On the other hand, two patients with induced OMR but unfavourable outcome (see Table 4) did not show a dilated pupil. Accordingly, they would not have been identified if both OMR and pupillary size analysis were required for classification. This suggests that VOM may be the more sensitive indicator. The functional integrity of the brainstem could also be examined by the corneal reflex and by the gag reflex. But the patients here in the acute phase all were sedated, received analgetic therapy and were intubated. So in clinical investigation we find these reflexes suppressed or absent independent from the later outcome. In the later phases, when sedation and analgetic therapy is reduced for awakening, these reflexes could be of prognostic value.

As a predictor for an outcome the GCS scale indicates that low score values statistically correspond to high mortality [13]. But an individual prognosis cannot be given by the post-resuscitation GCS score. Different technologies are employed on the intensive care unit for multimodal monitoring, for example, intracerebral oxygen partial pressure [14], near infrared-spectroscopy [15] or microdialysis [16,17]. These reflect a local cerebral situation which can describe changes in the metabolism. Therefore multimodal monitoring is used to prevent secondary injury by specific therapy [18,19].

Advancement of TBI classification is recognized as one of the major goals in head injury research [20]. Thus, refined definition of injury patterns taking pathophysiological mechanisms and pathoanatomic conditions into consideration should improve the specific treatment appropriate to the individual case. TBI classification also has an impact on prognostic classification.

Prognosis of outcome in TBI is currently determined by various methods. All have their limitations and cannot always be employed during the acute post-traumatic phase. Here electro encephalogram (EEG) could play an important role. But analgesic and sedative therapy is the standard procedure in sTBI in the intubated and ventilated patient. Due to that medication the value of EEG for prognosis is clearly reduced. In this series burst suppression EEG was used in some patients for monitoring the barbiturate narcosis.

Evoked potentials (EP) also play a valuable role in assessing prognosis in the early post-traumatic phase [21-24]. It has been demonstrated that pathological findings in somatosensory EP are closely linked to poor outcome. However, EPs are currently used for a patient's classification, which alone does not considerably influence the neurotraumatologic management [25].

In general, the practical use of EPs is subject to various limitations. Acoustic EPs can be influenced by impaired transduction due to pre-existing or traumatic audiological pathologies. Motor- and somatosensensory EPs rely on the exclusion of efferent and afferent nerve and plexus lesion. It may in future be instructive to compare the results of VOM with those determined by sensory and acoustic EPs [26,27] or with those from vestibular evoked myogenic potential (VEMP) and ocular vestibular evoked myogenic potentials [28]. The comparison of the predictive values of VOM and these methods should be addressed in further studies.

Vestibulo-ocular testing in comatose patients has been used in the past [29-31], including caloric testing of the VOR, which has been widely use. However, water irrigation cannot be employed in the presence of lacerations of the membrana tympani or by haemorrhage clots in the meatus acusticus externus which are common. It is imperative that the meatus is inspected, and in many cases an ear, nose and throat (ENT) specialist should be consulted before caloric testing is performed. Vestibulo-ocular testing based on oculo-cephalic movement requires an intact cervical spine and an intact cervico-occipital junction [32]. This excludes its use in cases of TBI confounded by spinal injury. In the present study, six patients (22%) had suffered spinal injury, five of which were located in the cervical or thoracic segments, excluding them from such manoeuvres. The use of VOM avoids the limitations of caloric and oculo-cephalic testing. Trauma to external ear or to the membrana tympani have no influence on the galvanic stimulus, which acts directly on the vestibular nerve. Furthermore, no physical manipulation of the patient is required. The electrodes and VOG device can be mounted with the patient in the supine position without any repositioning manoeuvres. The technique is therefore applicable in cases with spinal trauma. The time required for bedside examination is of the order of a few minutes. Stimulus parameters (frequency and amplitude) are defined and the GaLa stimulation can be repeated as necessary. The video recording of the OMR provides documentation and permits offline analysis of the OMR. Thus in the present study, cases were identified where the OMR was too small to be seen by the naked eye. The correlation of eye movement with the GaLa stimulus was revealed only after objective analysis of the video recordings, which included evaluation of the three-dimensional (that is, horizontal, vertical and torsional) eye movement response.

In addition to the use of functional testing as a means for assessing patients' outcome after TBI, imaging techniques are used. Considering structural lesions detected by cranial computer tomography (CCT) in both outcome groups (favourable and unfavourable) the complete spectrum of trauma was detected (Table 2).

A formal classification of CCT findings for outcome prognosis in sTBI, including brain shift, compression of cisterns and size of haematoma has been implemented by Marshall [33]).

These structural lesions included in that classification does not allow giving a individual prognosis in many cases. This could be due to the difficulty in detecting diffuse axonal injury [34,35] and lesions of the brainstem [2].

Recently, research into the value of imaging for prognosis has focussed on the brainstem. Here, Magnetic Resonance Imaging (MRI) studies have shown a high correlation between brainstem lesion and poor outcome, and have resulted in a classification system [2]. Thus, it would be of interest to perform a comparative study of MRI and VOM in TBI patients.

Mannion [36] describes brainstem lesions seen in MRI after sTBI which were not connected to a poor outcome; further, not all patients with unfavourable outcome showed any brainstem lesion. Thus, a lesion-free or lesioned brainstem, as determined by MRI, does not provide a reliable indicator for outcome. Carpentier [37] describes 'invisible brainstem damage' in MRI, which was further characterized and better correlated to patients with poor outcome using magnetic resonance spectroscopy. Accordingly, a clear separation between GOS 1 to 2, GOS 3 and GOS 4 to 5 was made possible by combining metabolic (spectroscopy) and anatomic (imaging) brainstem data.

It remains, however, that MRI scanning in TBI patient involves a number of technical restrictions, including the need for non-magnetic equipment, positioning tolerance of the patient with regard to the intracranial pressure and the complexity of patient transportation. These are no longer an issue when using VOM. Thus, VOM represents a practicable, complementary technique for the evaluation of outcome in comatose patients.

## Conclusions

It was possible to predict patients' outcomes by distinguishing two groups using VOM in the acute phase of sTBI. As an indicator of brainstem function VOM provides a useful, complementary approach to the identification of brainstem lesions by imaging techniques.

## Key messages

• Already in the acute phase after TBI, VOM is useful to predict patient's outcome. This prediction permits a distinction between an outcome of GOS</=2 or GOS>2 with high significance (two-sided Fisher-Test *P*<0.001).

• VOM can be applied on the ICU ward with high reliability and without high effort. VOM is not influenced by sedative medication.

## Abbreviations

APACHE II: The Acute Physiology And Chronic Health Evaluation; CCT: cranial computer tomography; CT: computer tomography; EEG: Electro encephalogram; EP: evoked potentials; ENT: ear nose and throat department; FFT: Fast Fourier Transformation; GaLa: Galvanic labyrinth polarization; GCS: Glasgow Coma Score; GOS: Glasgow Outcome Score; icp: intracranial pressure; MRI: Magnetic Resonance Imaging; nicu: neuro intensive care unit; OMR: oculomtor response; SAPS: Simplified Acute Physiology Score; sTBI: severe traumatic brain injury; VEMP: vestibular evoked myogenic potential; VOG: video-oculography; VOM: vestibulo-ocular monitoring; VOR: vestibulo-ocular reflex.

## Competing interests

The authors declare that they have no competing interests. This study was supported by university research grants from the Charité (research commission and committee for young scientists). This novel VOM technique was recently patented.

## Authors' contributions

HGS developed VOM, designed the study and performed the examinations and contributed as principle investigator. JNL contributed in the technical set up design of VOM and contributed to the data analysis. PV participated in the design of the study, evaluated results in clinical context and revised the manuscript. AHC contributed to the experimental set-up, the data analysis and drafted the manuscript.

## References

[B1] Firsching R Woischneck D Diedrich M Klein S Ruckert A Wittig H Dohring W Early magnetic resonance imaging of brainstem lesions after severe head injury J Neurosurg 1998 89 707 712 10.3171/jns.1998.89.5.0707 9817405

[B2] Firsching R Woischneck D Klein S Reissberg S Dohring W Peters B Classification of severe head injury based on magnetic resonance imaging Acta Neurochir (Wien) 2001 143 263 271 10.1007/s007010170106 11460914

[B3] Firsching R Woischneck D Klein S Ludwig K Dohring W Brain stem lesions after head injury Neurol Res 2002 24 145 146 10.1179/016164102101199684 11877897

[B4] Schlosser HG Unterberg A Clarke A Using video-oculography for galvanic evoked vestibulo-ocular monitoring in comatose patients J Neurosci Methods 2005 145 127 131 10.1016/j.jneumeth.2004.12.004 15922031

[B5] Galvani L Il 'Taccuino'; Riproduzione in fac-simile dell'autografo conservato nella Biblioteca dell'Archiginnasio di Bologna A cura del Comitato per la celebrazione del 2 centenario della nascita di L. Galvani 1937 Bologna: Zanichelli

[B6] Goldberg JM Fernandez C Smith CE Responses of vestibular-nerve afferents in the squirrel monkey to externally applied galvanic currents Brain Res 1982 252 156 160 10.1016/0006-8993(82)90990-8 6293651

[B7] Goldberg JM Smith CE Fernandez C Relation between discharge regularity and responses to externally applied galvanic currents in vestibular nerve afferents of the squirrel monkey J Neurophysiol 1984 51 1236 1256 6737029 10.1152/jn.1984.51.6.1236

[B8] Smith CE Goldberg JM A stochastic afterhyperpolarization model of repetitive activity in vestibular afferents Biol Cybern 1986 54 41 51 10.1007/BF00337114 3487348

[B9] Schlosser H-G Guldin WO Grüsser O-J Tuning in caudal fastigial nucleus units during natural and galvanic labyrinth stimulation Neuroreport 2001 12 1443 1447 10.1097/00001756-200105250-00029 11388426

[B10] Schlosser H-G Guldin WO Fritzsche D Clarke A Transcranial Doppler ultrasound depicts central vestibular processing in galvanic labyrinth polarization - demonstrating bilateral vestibular projection Eur J Neurosci 2008 28 372 378 10.1111/j.1460-9568.2008.06331.x 18702708

[B11] Schlosser H-G Guldin WO Evidence for vestibular processing using vector addition in caudal fastigial nucleus Med Sci Monit 2009 14 19396045

[B12] Schlosser H-G Guldin WO Fritzsche D Clarke A Transcranial Doppler ultrasound depicts central vestibular processing in galvanic labyrinth polarization - demonstrating bilateral vestibular projection European Journal of Neuroscience 2008 28 372 378 10.1111/j.1460-9568.2008.06331.x 18702708

[B13] Marshall LF Gautille T Klauber M The outcome of severe closed head injury J Neurosurg 1991 75 S28 S36

[B14] Meixensberger J Xenon 133--CBF measurements in severe head injury and subarachnoid haemorrhage Acta Neurochir Suppl (Wien) 1993 59 28 33 7906078 10.1007/978-3-7091-9302-0_5

[B15] Kirkpatrick PJ Smielewski P Czosnyka M Menon DK Pickard JD Near-infrared spectroscopy use in patients with head injury J Neurosurg 1995 83 963 970 10.3171/jns.1995.83.6.0963 7490639

[B16] Bullock R Zauner A Woodward JJ Myseros J Choi SC Ward JD Marmarou A Young HF Factors affecting excitatory amino acid release following severe human head injury J Neurosurg 1998 89 507 518 10.3171/jns.1998.89.4.0507 9761042

[B17] Hillered L Valtysson J Enblad P Persson L Interstitial glycerol as a marker for membrane phospholipid degradation in the acutely injured human brain J Neurol Neurosurg Psychiatry 1998 64 486 491 10.1136/jnnp.64.4.486 9576540 PMC2170060

[B18] Bouma GJ Muizelaar JP Choi SC Newlon PG Young HF Cerebral circulation and metabolism after severe traumatic brain injury: the elusive role of ischemia J Neurosurg 1991 75 685 693 10.3171/jns.1991.75.5.0685 1919689

[B19] Chesnut RM Marshall LF Klauber MR Blunt BA Baldwin N Eisenberg HM Jane JA Marmarou A Foulkes MA The role of secondary brain injury in determining outcome from severe head injury J Trauma 1993 34 216 222 10.1097/00005373-199302000-00006 8459458

[B20] Saatman KE Duhaime AC Bullock R Maas AI Valadka A Manley GT Classification of traumatic brain injury for targeted therapies J Neurotrauma 2008 25 719 738 10.1089/neu.2008.0586 18627252 PMC2721779

[B21] Rappaport M Hall K Hopkins K Belleza T Berrol S Reynolds G Evoked brain potentials and disability in brain-damaged patients Arch Phys Med Rehabil 1977 58 333 338 880010

[B22] Greenberg RP Mayer DJ Becker DP Miller JD Evaluation of brain function in severe human head trauma with multimodality evoked potentials. Part 1: Evoked brain-injury potentials, methods, and analysis J Neurosurg 1977 47 150 162 10.3171/jns.1977.47.2.0150 874542

[B23] Greenberg RP Becker DP Miller JD Mayer DJ Evaluation of brain function in severe human head trauma with multimodality evoked potentials. Part 2: Localization of brain dysfunction and correlation with posttraumatic neurological conditions J Neurosurg 1977 47 163 177 10.3171/jns.1977.47.2.0163 874543

[B24] Riffel B Kroiß H Stöhr M Diagnostik und Prognostik mit Evozierten Potentialen in der Intensivmedizin 1994 Stuttgart, Berlin, Köln: Kohlhammer

[B25] Marshall LF Marshall SB Outcome prediction in severe head injury 1996 New York, St. Louis, San Francisco: McGraw-Hill

[B26] Gaetz M The neurophysiology of brain injury Clin Neurophysiol 2004 115 4 18 10.1016/S1388-2457(03)00258-X 14706464

[B27] Amantini A Grippo A Fossi S Cesaretti C Piccioli A Peris A Ragazzoni A Pinto F Prediction of 'awakening' and outcome in prolonged acute coma from severe traumatic brain injury: evidence for validity of short latency SEPs Clin Neurophysiol 2005 116 229 235 10.1016/j.clinph.2004.07.008 15589201

[B28] Todd NP Roesengren SM Aw ST Colebatch JG Ocular vestibular myogenic potentials (OVEMPs) produced by air- and bone-conducted sound Clin Neurphysiol 2007 118 381 390 10.1016/j.clinph.2007.05.068 17141563

[B29] Buettner UW Ocular motor dysfunction in stupor and coma Baillieres Clin Neurol 1992 1 289 300 1344071

[B30] Toglia JU Adam RU Stewart G Galvanic vestibular tests in the assessment of coma and brain death Ann Neurol 1981 9 294 296 10.1002/ana.410090314 7224591

[B31] Mueller-Jensen A Neunzig HP Emskotter T Outcome prediction in comatose patients: significance of reflex eye movement analysis J Neurol Neurosurg Psychiatry 1987 50 389 392 10.1136/jnnp.50.4.389 3585347 PMC1031870

[B32] Buettner UW Zee DS Vestibular testing in comatose patients Arch Neurol 1989 46 561 563 2653293 10.1001/archneur.1989.00520410095030

[B33] Marshall LF Marshall SB Klauber MR Van Berkum Clark M Eisenberg H Jane JA Luerssen TG Marmarou A Foulkes MA The diagnosis of head injury requires a classification based on computed axial tomography J Neurotrauma 1992 9 Suppl 1 S287 292 1588618

[B34] Gennarelli TA Thibault LE Adams JH Graham DI Thompson CJ Marcincin RP Diffuse axonal injury and traumatic coma in the primate Ann Neurol 1982 12 564 574 10.1002/ana.410120611 7159060

[B35] Adams JH Doyle D Graham DI Lawrence AE McLellan DR Gennarelli TA Pastuszko M Sakamoto T The contusion index: a reappraisal in human and experimental non-missile head injury Neuropathol Appl Neurobiol 1985 11 299 308 10.1111/j.1365-2990.1985.tb00027.x 4058674

[B36] Mannion RJ Cross J Bradley P Coles JP Chatfield D Carpenter A Pickard JD Menon DK Hutchinson PJ Mechanism-based MRI classification of traumatic brainstem injury and its relationship to outcome J Neurotrauma 2007 24 128 135 10.1089/neu.2006.0127 17263676

[B37] Carpentier A Galanaud D Puybasset L Muller JC Lescot T Boch AL Riedl V Cornu P Coriat P Dormont D van Effenterre R Early morphologic and spectroscopic magnetic resonance in severe traumatic brain injuries can detect "invisible brain stem damage" and predict "vegetative states" J Neurotrauma 2006 23 674 685 10.1089/neu.2006.23.674 16689669

